# Harmonization of social and physical health measures across prospective clinical studies of combat exposed service members and veterans: the total brain diagnostics program

**DOI:** 10.3389/fneur.2026.1799509

**Published:** 2026-07-08

**Authors:** Shannon M. Nugent, Kate Clauss, Sara Vlajic, Maya E. O’Neil, Samuel R. Walton, Kelly M. Reavis, Christine Clermont, Alyssa Currao, Cree Foeller, Catherine B. Fortier, Sreekanth Kamineni, Arielle R. Knight, Aubrey A. Knoff, Landon B. Lempke, Helal Mobasher, William C. Walker, William P. Milberg, David X. Cifu

**Affiliations:** 1 Center to Improve Veteran Involvement in Care, Veterans Affairs Portland Health Care System, Portland, OR, United States; 2 Department of Psychiatry, Oregon Health & Science University, Portland, OR, United States; 3 Northwest Mental Illness Research, Education, and Clinical Center (MIRECC), Veterans Affairs Portland Health Care System, Portland, OR, United States; 4 Department of Physical Medicine and Rehabilitation, Virginia Commonwealth University School of Medicine, Richmond, VA, United States; 5 Institute for Women's Health, Virginia Commonwealth University, Richmond, VA, United States; 6 Richmond Veterans Affairs Medical Center, Central Virginia Veterans Affairs Health Care System, Richmond, VA, United States; 7 Translational Research Center for TBI and Stress Disorders National Network Center, VA Boston Healthcare System, Boston, MA, United States; 8 National Center for Rehabilitative Auditory Research (NCRAR), Veterans Affairs Portland Health Care System, Portland, OR, United States; 9 National Center for PTSD, VA Boston Healthcare System, Boston, MA, United States; 10 Department of Psychiatry, Harvard Medical School, Boston, MA, United States; 11 Department of Psychiatry, Boston University Chobanian and Avedisian School of Medicine, Boston, MA, United States; 12 University of Utah School of Medicine, Salt Lake City, UT, United States; 13 Salt Lake City Veteran Affairs Medical Center, Salt Lake City, UT, United States; 14 Department of Psychological and Brain Sciences, Boston University, Boston, MA, United States; 15 Sheltering Arms Institute, Richmond, VA, United States; 16 Geriatric Research, Education and Clinical Center, VA Boston Healthcare System, Boston, MA, United States

**Keywords:** data harmonization, physical health, psychosocial health, traumatic brain injury, veteran health

## Abstract

**Introduction:**

Traumatic Brain Injury is prevalent during military service and is associated with short- and long-term psychosocial and functional changes, though comprehensive longitudinal data on Veterans and active-duty service members (SMs) are lacking. To address this research gap, we utilized data from the Long-term Impact of Military-relevant Brain Injury Consortium (LIMBIC) and the Translational Research Center for TBI and Stress Disorders (TRACTS), two prospective longitudinal cohort studies of veterans and active duty SMs, each containing a wide range of symptom scales, objective assessments, and health-related outcomes.

**Methods:**

This paper describes the innovative methods used to achieve the initial proof-of-concept harmonization for baseline psychosocial and physical health data from these two large cohort studies. To achieve harmonization, we gathered a multidisciplinary team with clinical and research expertise. We created a list of measures utilized by each study and organized them into larger clinically meaningful domains. When possible, we harmonized full measures, or single items directly, while others needed to be indirectly harmonized, by recoding, aligning categorical levels, and categorizing scales based on established cut scores. We calculated descriptive statistics to summarize and compare data. We then conducted Principal Component Analysis (PCA) for all continuous measures to assess whether site-level effects were observed in the shared variance.

**Results:**

A total of 73 variables capturing psychosocial function, sensorimotor, pain, and clinical health factors were harmonized across the LIMBIC and TRACTS studies. There were no differences in sex or ethnicity distributions between the studies. Sensory, social health, and health related clinical data were broadly comparable across cohorts, while pain intensity and headache disability were higher in LIMBIC. PCA analysis suggests data is suitable for pooled analysis.

**Discussion:**

We were able to directly harmonize multiple self-report measures of social well-being and indirectly harmonize other functional and demographic variables. While this initial effort focused on baseline data, the included principles can be employed to harmonize longitudinal data to increase the ability to detect clinical phenotypes to be applied in precision medicine approaches in future research.

## Introduction

Traumatic Brain Injury (TBI) is a priority research area among veterans and military service members (SMs) due to its high prevalence during their military careers (1). Depending on the severity, individuals with TBI often have many short- and long-term symptoms, psychosocial issues and functional changes (2). To date, cross-sectional and longitudinal studies have examined post-TBI psychosocial and functional outcomes including social functioning, neurobehavioral functioning, sensorimotor functioning, pain (3), sleep (4, 5), employment status, and satisfaction (6) with life (7–10). To gain a more nuanced understanding of how TBI may differentially affect individuals, some studies have examined population-based differences among psychosocial and physical outcomes (9–13). These more nuanced studies have been limited to small sample sizes or cross-sectional examinations, hindering the ability to detect specific phenotypes or temporal associations of TBI and subsequent measures of functioning. There are several extant longitudinal studies on TBI outcomes, including TRACK-TBI (14) and the Traumatic Brain Injury Model Systems (TBIMS) Program (15) which offer insights into temporal trends between a TBI event and longer term functional and psychosocial outcomes. TBIMS program takes this even a step further by offering methodological consultation and data infrastructure to 16 different sites who are collecting longitudinal outcomes data to enhance data collection rigor and efficiency. Yet, both of these important longitudinal studies are not focused on Veteran or military service members and do not include efforts to harmonize a combination of biological, psychological and social variables across different studies. Additionally, TBIMS is restricted to moderate to severe TBI requiring inpatient rehabilitation (IPR) and therefore excludes patients with mild TBI or even more severe TBI who do not enter IPR. The lack of such detailed data to inform targeted, personalized treatments and interventions presents an ongoing challenge for managing the myriad clinical presentations of TBI among Veterans and active duty SMs.

To address this research gap, this project utilized data from the Long-term Impact of Military-relevant Brain Injury Consortium (LIMBIC) and the Translational Research Center for TBI and Stress Disorders (TRACTS) (16, 17), two separate, prospective longitudinal cohort studies of veterans and active duty SMs. These studies provide deep characterizations of individuals with and without history of TBI through robust clinical research methodologies. While both studies include comparator participants with no history of TBI, LIMBIC enrolls only those with mild TBI, while TRACTS enrolls those with predominantly mild TBI histories, though does not exclude participants with moderate and severe TBI history. LIMBIC and TRACTS both evaluate a wide range of symptom scales, objective assessments, health-related physical outcome measures, and both modifiable and non-modifiable health factors that may influence or measure outcomes or care needs. Many of these assessments and collection methods overlap between LIMBIC and TRACTS, presenting a unique opportunity to harmonize across studies to enhance the sample size and power to assess temporal relationships, subgroup differences, and modifiable risk factors of brain health among SMs and veterans with history of mild TBI.

Pooling participant data from these large, longitudinal TBI research studies with current and former military personnel will yield an extensive dataset enabling more focused analyses on specific subgroups or less common outcomes, while also reflecting a more holistic generalizable population estimates of SMs and veterans ultimately leading to more impactful and clinically informative findings. Specifically, identifying the temporal relationships between psychosocial and physical functioning with individual health characteristics, TBI symptom burden, and response to interventions can enhance our understanding of the clinical application of these testing tools. While the Total Brain Diagnostics (TBD) Program overview and harmonization of patient and TBI characteristics across LIMBIC and TRACTS has been published (18), this paper describes the innovative methods used by the TBD program, to achieve the initial proof-of-concept harmonization approach to the baseline social and physical health-related variables.

## Materials and methods

### Databases

Since 2013, the LIMBIC (Prospective Longitudinal Study [PLS]) has followed a large cohort of combat-exposed veterans and SMs residing across the continental U. S. through annual examinations across 10 geographically dispersed sites (16). As of September 2025, 3,012 participants have enrolled and completed their initial assessment battery. Since 2009, TRACTS has followed a similar large cohort of combat exposed U. S. military SMs and veterans enrolled for baseline, with follow-ups at 2-years and every 5-years thereafter in an ongoing 15-year, two-site (Boston and Houston), longitudinal study (17). As of September 2025, more than 1,070 participants have completed baseline assessments.

LIMBIC and TRACTS both evaluate a wide range of symptom scales, objective assessments, health-related outcome measures, and both modifiable and non-modifiable factors that may influence or measure outcomes or care needs including sociodemographic information, TBI characteristics, clinical examinations, neurocognitive assessments and a breadth of functional measures. Each study also includes biomarker data, advanced neuroimaging, electroencephalography, and computerized eye tracking.

### Total brain diagnostics project overview

The TBD program aims to test proof-of-concept approaches to harmonize identical and common construct data elements of the LIMBIC and TRACTS studies to build a single, integrated dataset to be used for more comprehensive analyses. The overarching goal was to demonstrate feasibility of harmonization approaches across clinical and biometric data from these two deeply characterized cohorts (18). For the present manuscript, we included initial (baseline) visit data for social and physical health-related variables that could be harmonized. We selected physical and social well-being as two key facets of individual health as defined by the World Health Organization (19).

### Data harmonization

#### Harmonized measures

**Figure 1:**
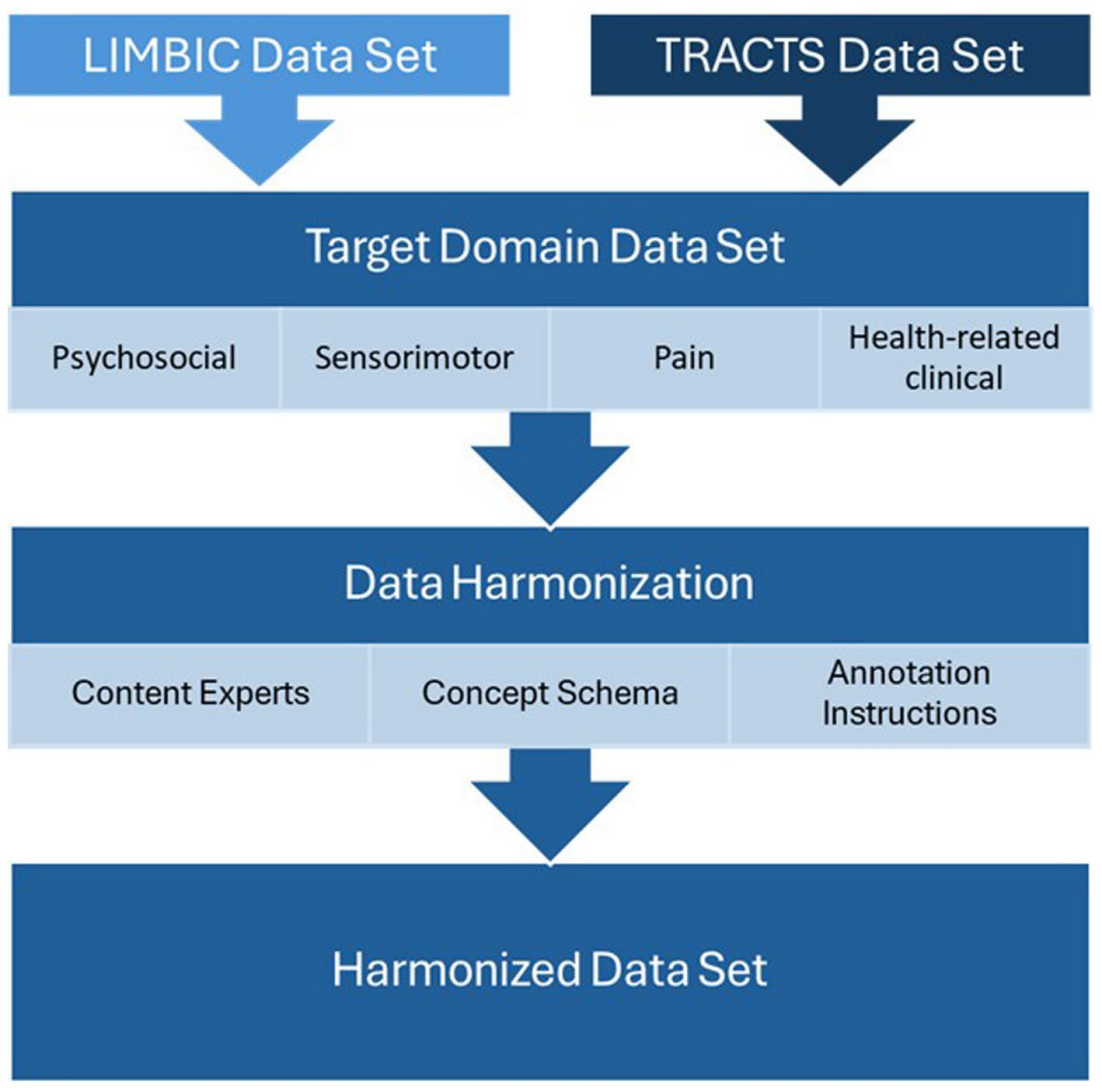
Overview of data source and harmonization process.

Though assessments and variables in the LIMBIC and TRACTS studies were identical in many cases, harmonization was necessary to develop a valid combined dataset (see Figure 1). To harmonize the data, we brought together investigators across the LIMBIC and TRACTS projects with expertise in physical and mental health, kinesiology, epidemiology, neuropsychology, neuroimaging, bioinformatics, quantitative data analytics, and data management. Through regular expert team meetings, we first identified several overlapping subdomains of patient-centered assessments, including social, sensory, pain, and health-related clinical measures. Data dictionaries for both studies were reviewed to ensure a comprehensive understanding of study variables and inform as preliminary crosswalk within each domain (see Supplementary Figure S1). Next, we identified measures with an exact match in both studies (e.g., The Pittsburgh Sleep Quality Index - PSQI) that could be harmonized at the item level as well as measures that generated similar variables that could be operationally harmonized in a nearly synonymous manner (e.g., Likert pain scale ranging from 0 to 10 within the past week, extracted from separate measures in each study). For domains of interests that did not have an exact match, we chose the most comparable variables based on item content, established crosswalks, or published cut scores indicating clinical significance, prioritizing harmonization approaches that retained the continuous nature of the data whenever possible. All decisions were made collaboratively within the expert workgroup and cleared by the leadership team. All measures listed below were administered in person as part of the LIMBIC and TRACTS assessment batteries and scored according to published instructions. In addition to the aforementioned clinical subdomains, measures listed below are organized by those that could be directly harmonized and those that could be indirectly or partially harmonized. Any deviations are noted.

##### Directly harmonized measures

###### Social health

*Deployment Risk and Resilience Inventory-2 Post Deployment Social Support Scale.* The Post Deployment Social Support scale, a subscale of the 17-measure Deployment Risk and Resilience Inventory-2, uses self-report Likert scales (i.e., 1 = “Strongly disagree” to 5 = “Strongly agree”) to assess perceived social–emotional support (e.g., feeling understood by others, levels and quality of companionship, etc.) and instrumental tangible assistance (e.g., help with tasks of daily living, offering financial support etc.) (20). DRRI Social Support Total Scores range from 10 to 50.

*Satisfaction with Life Scale (SWLS).* The SWLS is a 5-item questionnaire that measures overall life satisfaction. The SWLS uses a self-report 7-point Likert scale, with response anchors, 1 = “Strongly disagree” to 7 = “Strongly agree” to questions like “In most ways my life is close to ideal” and “So far I have gotten the most important things I want in life.” Total scores range from 5 (low satisfaction) to 35 (high satisfaction) (21).

###### Sensory

*Dizziness Handicap Inventory Screening (DHI-S).* The DHI-S is a 10-item screening version of the longer DHI used to determine the physical, emotional, and functional impacts of dizziness on a person’s life. Respondents answer “Yes” (4) or “No” (0) or “Sometimes” (2) for each item. Total scores on the DHI-S range of 0 to 40 (22). DHI-S total scores were categorized into little or no disability (0–10), mild disability (12-20), moderate disability (22-30), and severe disability (32-40) (23).

*Hearing Handicap Inventory for Adults Screening (HHIA-S).* The HHIA-S is an abbreviated version of the longer HHIA. The 10-question self-report assessment is composed of emotional and social subscales related to hearing impairment and is scored on a range of 0 to 40 (24). Respondents answer “Yes” (4) or “No” (0) or “Sometimes” (2) for each item. HHIA-S total scores were categorized as little or no disability (0–8), mild or moderate disability (9–25), and severe disability (26–40) (25).

*Presence of tinnitus in last week.* This is a single self-report item that assesses whether a participant experienced ringing in their ears during the past 7 days (yes/no).

*Other Sensory Symptoms:* Several individual items from the self-report Neurobehavioral Symptom Inventory (NSI) (26) captured additional sensory experiences in both LIMBIC and TRACTS. These items included: Feeling Dizzy; Loss of balance; Poor coordination, clumsy; Nausea, Vision problems, blurring, trouble seeing; Sensitivity to light; Sensitivity to noise; Numbness or tingling on parts of my body; Change in taste and/or smell, and loss of appetite or increased appetite. The NSI items use a 5-point Likert scale from 0 to 4, categorized as none, mild, moderate, severe, and very severe.

###### Pain

*Brief Pain Inventory (BPI)* and *NIH Toolbox:* Pain in last week. Pain during the previous week was measured in two ways. TRACTS utilized the BPI short form (27), a 9-item scale, with item response ranging from 0 (no pain) to 10 (worst pain imaginable), with one question inquiring about the severity of pain in last week. LIMBIC utilized the National Institute of Health (NIH) Toolbox Pain subscale (28), 10-item scale, with item responses also ranging from 0 (no pain) to 10 (worst possible pain), that includes one question assessing severity of pain during the prior week. On both measures, ‘worst pain in the past week’ is reported on a scale of 0 to 10, with higher scores indicating worse pain, which was the item that was harmonized.

###### Health-related clinical

*Sleep Quality: Pittsburgh Sleep Quality Index (PSQI).* The PSQI is a 19-item questionnaire designed to measure sleep quality and disruptions over a 1-month interval (29). Items load on to 7 components (e.g., sleep duration, sleep disturbance), which are scored 0–3 resulting in a total score range from 0 to 21, where higher scores indicate poorer sleep quality and scores of greater than 5, suggest sleep difficulties.

*Blood Pressure.* Blood pressure is a measure of a person’s diastolic and systolic blood pressure taken at rest while seated (LIMBIC) both seated and standing (TRACTS) during a physical exam in which measurement was performed manually and automatically using a blood pressure cuff for each participant.

*Heart Rate.* Heart rate is the number of times a person’s heart beats per minute and was collected while seated (LIMBIC) and standing during a physical exam in which measurement was performed manually and automatically for each participant.

*Fatigue.* Fatigue, loss of energy, getting tired easily, or drowsiness was assessed using a single item from the NSI. This item was self-reported on a 5-point Likert scale from 0 to 4, categorized as none, mild, moderate, severe, and very severe.

##### Indirect or partially harmonized measures

###### Social health

*Employment.* Employment was assessed using a self-report question about the types of jobs the participant has possessed (including paid or volunteer). TRACTS participants reported their current employment status, choosing from the options: not working, part-time, or full-time. LIMBIC inquired whether participants were employed and if so, the number of paid hours per week worked in the past month. To align with TRACTS classifications, hours worked were categorized as part-time for participants who worked fewer than 35 h per week and full-time for those who worked 35 h (30) or more per week.

###### Pain

*Headache Disability.* This measure describes the impact of migraines and headaches on a participant’s life. In the TRACTS study, the impact of headaches was assessed using Migraine Disability Assessment (MIDAS), a 5-item self-report scored 0–21+ (31). MIDAS total scores were categorized into little or no disability (0–5), mild disability (6-10), moderate disability (11-20), and severe disability (≥ 21). In LIMBIC, the team used the Headache Impact Test – 6 (HIT-6), which is a 6-item self-report ranging of 36–78 to assess the impact of headaches on daily life (32) HIT-6 total scores were categorized into little or no disability (≤ 49), mild disability (50–55), moderate disability (56–59), and severe disability (60–78) (31).

###### Sensory

*Tinnitus disability.* Tinnitus disability was measured via the use of two indices: The Tinnitus Functional Index (TFI), used by LIMBIC (33), and the Tinnitus Handicap Inventory (THI), used by TRACTS (34), which are both 25 question forms scored on a 0–100 scale used to assess the impact of tinnitus on a person’s life. TFI total scores were categorized into little or no disability (0–17), mild disability (18-31), moderate disability (32–53), severe disability (54–72), and extremely severe disability (73–100). THI total scores were categorized into little or no disability (0–16), mild disability (18–36), moderate disability (38–56), severe disability (58–76), and extremely severe disability (78–100) (25).

###### Health-related clinical

*Body Mass Index (BMI).* Body Mass Index is a calculated measure that uses a person’s height and weight, collected during a physical exam (LIMBIC PLS) or self-report (TRACTS), to determine a person’s quantity of body fat (35).

###### Demographic information

Demographic information was self-reported via standardized intake forms for each separate study. Common information included age, biological sex, racial and ethnic identity.

### Data merging and quality assessment

When possible, direct matches between studies were merged one-to-one without adjustments to the merged data set. Direct variable matches but with coding differences were recoded to align (e.g., presence of tinnitus was recoded from 1 = “yes” and 2 = “no” to 1 = “yes” and 0 = “no”). Partial matches included variables that measured the same construct with different instruments, different versions of the same instrument, or different variable categories (e.g., employment). Harmonization strategies were applied based on the type of partial match. For variables that used different instruments (e.g., MIDAS and HIT-6) continuous scores were converted into categorical variables based on clinical cut scores described above. When a short form was used (e.g., DHI and DHI-S) only items shared by both versions were used to calculate the total scores. To maximize complete-case data across sites to conduct Principal Component Analysis (PCA), we imputed a value of zero for HIT-6, DHI-S and HHIA-S total scores in cases where participants did not complete the full questionnaire as a result of precursor screening procedures that determined whether or not the measures were administered. For example, if an individual denied experiencing any recent headache, they were not administered the full instrument evaluating headache impact and the summary value for that measure was assumed to be 0 (no impact) and imputed as such. The ordered categorical headache and tinnitus disability variables were treated as continuous to meet PCA assumptions.

Once harmonized, we conducted descriptive statistics and data visualizations to detect implausible values, unexpected missingness, and misaligned category labels. Data merging and quality checks were conducted in R Statistical Software (36), version 4.4.1.

### Data analysis

We calculated descriptive statistics for combined data set as well as the individual LIMBIC and TRACTS datasets to facilitate cross-study comparisons. Demographic characteristics, age, sex, race, and ethnicity, were compared using Welch’s independent t-test for continuous variables and Chi-square or Fisher’s exact test for categorical variables. We estimated reliability of directly harmonized scales within LIMBIC and TRACTS using McDonald’s omega (ω). We then conducted PCA for all multi-item continuous measures including Social Support (DRRI), Headache Disability, Tinnitus Disability, DHI-S, HHIA-S, Pain Rating, PSQI, BMI, BP Systolic, BP Diastolic, and Heart Rate to visually inspect homogeneity across the two studies and 12 sites (10 in LIMBIC and 2 in TRACTS). PCA is a dimensionality reduction technique that retains most of the variation in a dataset. The algorithm identifies principal components (PCs), which are linear combinations of the variables that capture the greatest amount of variation. PCs were retained for further exploration based on a cut-off point of 70% cumulative variation explained (37). A correlation plot of the PCs and the harmonized variables was created to understand which variables contributed the most to variability in the dataset. The strength of correlations between variables and PCs was interpreted using the following criteria: weak (|r| = 0.1–0.3), moderate (|r| = 0.4–0.6), and strong (|r| = 0.7–1.0). The first two PCs were visualized in a score plot with 95% confidence interval ellipses and color-coded by site to identify distinct clustering. Overlapping points and ellipses suggest the multivariate data distributions are similar across sites.

## Results

A total of 73 variables capturing psychosocial function, sensorimotor, pain, and clinical health factors were overlapping enough to be harmonized across the LIMBIC and TRACTS studies. Of these, 33 had an exact match across studies and 34 variables had a “direct comparator,” meaning that items had originated from slightly different forms or data collection processes (e.g., self-reported weight vs. weight measured during a medical examination) but were able to be directly harmonized because all information was preserved. An additional 6 variables were successfully harmonized through a derived process using standard scores and/or equivalent clinical interpretation criteria.

There were no differences in sex or ethnicity distributions between the studies (*p* > 0.05, Table 1). However, there were significant differences in both age and race. The LIMBIC sample had a higher mean age of 43 years (SD = 10.5) at the initial assessment compared to TRACTS (*M* = 35, SD = 9.1). The LIMBIC sample also had slightly higher proportions of participants identifying as Black, White, or another racial identity. The two study samples had comparable proportions of participants identifying as American Indian or Alaska Native, Asian, Native Hawaiian or other Pacific Islander, and two or more racial identities.

**Table 1:** Demographic characteristics of LIMBIC and TRACTS participants by TBI history.

Characteristics	**LIMBIC** *N* = 2,868*^1^*		**TRACTS** *N* = 905*^1^*	
	**mTBI** *N* = 2,339*^1^*	**No TBI** *N* = 529*^1^*	**mTBI** *N* = 640*^1^*	**No TBI** *N* = 265*^1^*
Age	42.9 (10.5)	43.9 (11.6)	35.3 (9.1)	36.2 (10.2)
Sex				
Female	242 (10.3%)	112 (21.2%)	55 (8.6%)	45 (17.0%)
Male	2,095 (89.6%)	417 (78.8%)	585 (91.4%)	220 (83.0%)
Prefer not to answer/Unknown	2 (0.1%)	0 (0.0%)	0 (0.0%)	0 (0.0%)
Race				
American Indian or Alaska Native	20 (0.9%)	4 (0.8%)	6 (0.9%)	2 (0.8%)
Asian	37 (1.6%)	14 (2.6%)	11 (1.7%)	6 (2.3%)
Black	384 (16.8%)	120 (22.7%)	84 (13.1%)	47 (17.7%)
Native Hawaiian or Pacific Islander	15 (0.6%)	3 (0.6%)	4 (0.6%)	3 (1.1%)
White	1,677 (71.7%)	346 (65.4%)	443 (69.2%)	164 (61.9%)
Two or more races	110 (4.7%)	21 (4.0%)	31 (4.8%)	17 (6.4%)
Another race	82 (3.5%)	17 (3.2%)	11 (1.7%)	3 (1.1%)
Prefer not to answer/ Unknown	14 (0.6%)	4 (0.8%)	50 (7.8%)	23 (8.7%)
Ethnicity				
Hispanic or Latino	399 (17.1%)	91 (17.2%)	109 (17.0%)	64 (24.2%)
Not of Hispanic or Latino	1,909 (81.6%)	432 (81.7%)	521 (81.4%)	199 (75.1%)
Prefer not to answer/ Unknown	31 (1.3%)	6 (1.1%)	10 (1.6%)	2 (0.8%)

^1^Mean (SD); n (%).

### Descriptive results

To optimize comparability across LIMBIC and TRACTS cohorts, outcomes below are presented only for participants with no history of TBI or a history of one or more mTBI because LIMBIC does not enroll any cases of moderate or severe TBI (Tables 2–5).

**Table 2:** Descriptive statistics across the harmonized psychosocial function variables in LIMBIC and TRACTS by TBI History (no TBI versus Mild TBI).

**Characteristic**	**Harmony of scales**	**LIMBIC** *N* = 2,868		**TRACTS** *N* = 905	
		**mTBI** *n* = 2,339*^1^*	**No TBI** *n* = 529*^1^*	**mTBI** *n* = 640*^1^*	**No TBI** *n* = 265*^1^*
Psychosocial function					
Perceived social support (DRRI)	●	38.6 (8.1); 40.0 (34.0,45.0)	39.7 (7.7); 40.0 (35.0,46.0)	38.1 (8.3); 39.0 (32.0,45.0)	39.5 (8.7); 41.0 (34.0,46.0)
Unknown		29	2	349	140
Life satisfaction (SWLS)	●	21.3 (7.7); 22.0 (15.0,28.0)	22.4 (7.4); 23.0 (17.0,29.0)	20.3 (8.1); 20.0 (14.0,27.0)	22.7 (8.6); 25.0 (17.0,29.0)
Unknown		37	2	497	196
Employment	◐				
Full-time		1,262 (54.7%)	301 (57.2%)	315 (49.7%)	146 (55.5%)
Part-time		191 (8.3%)	56 (10.6%)	81 (12.8%)	33 (12.5%)
Not working		854 (37.0%)	169 (32.1%)	238 (37.5%)	84 (31.9%)
Unknown		32	3	6	2

^1^Mean (SD); Median (Q1, Q3); n (%). ● = exact match, ◐ = adjustments needed to harmonize ^a^TRACTS; ^b^LIMBIC-CENC; TBI = Traumatic Brain Injury, DRRI = Deployment Risk and Resilience Inventory, SWLS = Satisfaction with Life Scale.

**Table 3:** Descriptive statistics across the harmonized sensorimotor variables in LIMBIC and TRACTS by TBI History (no TBI versus Mild TBI).

Characteristic	Harmony of scales	LIMBIC *N* = 2,868		TRACTS *N* = 905	
		mTBI *n* = 2,339*^1^*	No TBI *n* = 529*^1^*	mTBI *n* = 640*^1^*	No TBI *n* = 265*^1^*
Sensorimotor					
Dizziness impact (DHI-S)	●				
Little or no disability		1,669 (72.3%)	474 (89.8%)	44 (75.9%)	21 (95.5%)
Mild disability		321 (13.9%)	25 (4.7%)	9 (15.5%)	1 (4.5%)
Moderate disability		215 (9.3%)	21 (4.0%)	5 (8.6%)	0 (0.0%)
Severe disability		105 (4.5%)	8 (1.5%)	0 (0.0%)	0 (0.0%)
Unknown		29	1	582	243
Hearing impact (HHIA-S)	●				
Little or No disability		1,261 (54.7%)	396 (75.0%)	79 (55.2%)	53 (79.1%)
Mild or moderate disability		720 (31.2%)	100 (18.9%)	44 (30.8%)	10 (14.9%)
Severe disability		324 (14.1%)	32 (6.1%)	20 (14.0%)	4 (6.0%)
Unknown		34	1	497	198
Tinnitus impact	◐ THI^a^ or TFI^b^				
Little or no disability		967 (44.6%)	337 (65.9%)	34 (34.7%)	15 (42.9%)
Mild disability		367 (16.9%)	68 (13.3%)	37 (37.8%)	10 (28.6%)
Moderate disability		525 (24.2%)	71 (13.9%)	16 (16.3%)	7 (20.0%)
Severe disability		309 (14.3%)	35 (6.8%)	11 (11.2%)	3 (8.6%)
Extremely severe disability		0 (0.0%)	0 (0.0%)	0 (0.0%)	0 (0.0%)
Unknown		171	18	542	230
NSI 1: Feeling dizzy	●				
None		1,014 (44.0%)	370 (70.1%)	302 (50.2%)	166 (64.6%)
Mild		818 (35.5%)	113 (21.4%)	171 (28.5%)	58 (22.6%)
Moderate		405 (17.6%)	39 (7.4%)	107 (17.8%)	29 (11.3%)
Severe		57 (2.5%)	6 (1.1%)	15 (2.5%)	4 (1.6%)
Very severe		12 (0.5%)	0 (0.0%)	6 (1.0%)	0 (0.0%)
Unknown		33	1	39	8
NSI 2: Loss of balance	●				
None		1,072 (46.5%)	367 (69.5%)	295 (49.2%)	185 (72.0%)
Mild		820 (35.5%)	124 (23.5%)	183 (30.5%)	51 (19.8%)
Moderate		339 (14.7%)	32 (6.1%)	97 (16.2%)	17 (6.6%)
Severe		65 (2.8%)	4 (0.8%)	20 (3.3%)	4 (1.6%)
Very severe		11 (0.5%)	1 (0.2%)	5 (0.8%)	0 (0.0%)
Unknown		32	1	40	8
NSI 3: Poor coordination, clumsy	●				
None		1,026 (44.5%)	360 (68.2%)	278 (46.3%)	167 (65.2%)
Mild		813 (35.3%)	129 (24.4%)	187 (31.1%)	55 (21.5%)
Moderate		395 (17.1%)	33 (6.3%)	102 (17.0%)	27 (10.5%)
Severe		60 (2.6%)	5 (0.9%)	30 (5.0%)	7 (2.7%)
Very Severe		12 (0.5%)	1 (0.2%)	4 (0.7%)	0 (0.0%)
Unknown		33	1	39	9
NSI 5: Nausea/vomiting	●				
None		1,499 (65.0%)	394 (74.8%)	443 (73.7%)	220 (85.6%)
Mild		498 (21.6%)	97 (18.4%)	90 (15.0%)	18 (7.0%)
Moderate		243 (10.5%)	29 (5.5%)	41 (6.8%)	13 (5.1%)
Severe		55 (2.4%)	7 (1.3%)	20 (3.3%)	5 (1.9%)
Very Severe		12 (0.5%)	0 (0.0%)	7 (1.2%)	1 (0.4%)
Unknown		32	2	39	8
NSI 6: Vision problems, blurring, trouble seeing	●				
None		957 (41.5%)	333 (63.2%)	324 (53.9%)	175 (68.1%)
Mild		810 (35.1%)	141 (26.8%)	143 (23.8%)	49 (19.1%)
Moderate		421 (18.2%)	41 (7.8%)	89 (14.8%)	23 (8.9%)
Severe		100 (4.3%)	9 (1.7%)	36 (6.0%)	8 (3.1%)
Very Severe		19 (0.8%)	3 (0.6%)	9 (1.5%)	2 (0.8%)
Unknown		32	2	39	8
NSI 7: Sensitivity to light	●				
None		806 (34.9%)	318 (60.2%)	249 (41.5%)	164 (63.8%)
Mild		621 (26.9%)	113 (21.4%)	131 (21.8%)	34 (13.2%)
Moderate		551 (23.9%)	55 (10.4%)	116 (19.3%)	37 (14.4%)
Severe		236 (10.2%)	38 (7.2%)	73 (12.2%)	14 (5.4%)
Very Severe		93 (4.0%)	4 (0.8%)	31 (5.2%)	8 (3.1%)
Unknown		32	1	40	8
NSI 9: Sensitivity to noise	●				
None		717 (31.1%)	264 (50.1%)	234 (39.0%)	147 (57.2%)
Mild		715 (31.0%)	141 (26.8%)	138 (23.0%)	39 (15.2%)
Moderate		596 (25.8%)	84 (15.9%)	129 (21.5%)	43 (16.7%)
Severe		219 (9.5%)	31 (5.9%)	72 (12.0%)	25 (9.7%)
Very severe		59 (2.6%)	7 (1.3%)	27 (4.5%)	3 (1.2%)
Unknown		33	2	40	8
NSI 10: Numbness or tingling on parts of my body	●				
None		666 (28.9%)	258 (48.9%)	255 (42.4%)	147 (57.6%)
Mild		748 (32.4%)	156 (29.5%)	150 (25.0%)	43 (16.9%)
Moderate		611 (26.5%)	82 (15.5%)	117 (19.5%)	41 (16.1%)
Severe		226 (9.8%)	25 (4.7%)	57 (9.5%)	20 (7.8%)
Very severe		56 (2.4%)	7 (1.3%)	22 (3.7%)	4 (1.6%)
Unknown		32	1	39	10
NSI 11: Change in taste and/or smell	●				
None		1,714 (74.3%)	452 (85.6%)	469 (78.0%)	215 (84.0%)
Mild		351 (15.2%)	47 (8.9%)	70 (11.6%)	23 (9.0%)
Moderate		178 (7.7%)	21 (4.0%)	40 (6.7%)	8 (3.1%)
Severe		52 (2.3%)	5 (0.9%)	14 (2.3%)	6 (2.3%)
Very severe		11 (0.5%)	3 (0.6%)	8 (1.3%)	4 (1.6%)
Unknown		33	1	39	9
NSI 12: Loss of appetite or increased appetite	●				
None		1,092 (47.3%)	342 (64.8%)	276 (46.0%)	156 (60.7%)
Mild		596 (25.8%)	115 (21.8%)	126 (21.0%)	41 (16.0%)
Moderate		442 (19.2%)	50 (9.5%)	122 (20.3%)	32 (12.5%)
Severe		140 (6.1%)	19 (3.6%)	56 (9.3%)	15 (5.8%)
Very Severe		38 (1.6%)	2 (0.4%)	20 (3.3%)	13 (5.1%)
Unknown		31	1	40	8

^1^Mean (SD); Median (Q1, Q3); n (%). ● = exact match, ◐ = adjustments needed to harmonize ^a^TRACTS; ^b^LIMBIC-CENC; HHIA-S = Hearing Handicap Inventory for Adults Screening, DHI-S = Dizziness Handicap Inventory Screening, THI = Tinnitus Handicap Index, TFI = Tinnitus Functional Index NSI = Neurobehavioral Symptom Inventory.

**Table 4:** Descriptive statistics across the harmonized pain variables in LIMBIC and TRACTS by TBI History (no TBI versus Mild TBI).

**Characteristic**	**Harmony of scales**	**LIMBIC** N = 2,868		**TRACTS** N = 905	
		**mTBI** *n* = 2,339*^1^*	**No TBI** *n* = 529*^1^*	**mTBI** *n* = 640*^1^*	**No TBI** *n* = 265*^1^*
**Pain**					
Pain intensity	● BPI and NIH Toolbox pain	4.3 (2.4); 4.0 (3.0,6.0)	3.2 (2.5); 3.0 (1.0,5.0)	6.2 (2.4); 7.0 (5.0,8.0)	5.7 (2.7); 6.0 (4.0,8.0)
Unknown		428	85	352	151
**Headache Impact**	◐ MIDAS^a^ or HIT-6^b^				
Little or no disability		914 (39.6%)	337 (64.1%)	23 (48.9%)	15 (65.2%)
Mild disability		305 (13.2%)	49 (9.3%)	5 (10.6%)	0 (0.0%)
Moderate disability		230 (10.0%)	30 (5.7%)	6 (12.8%)	7 (30.4%)
Severe disability		859 (37.2%)	110 (20.9%)	13 (27.7%)	1 (4.3%)
Unknown		31	3	593	242

^1^Mean (SD); Median (Q1, Q3); *n* (%), ● = exact match, ◐ = adjustments needed to harmonize ^a^TRACTS; ^b^LIMBIC-CENC; BPI = Brief Pain Inventory, NIH = National Institutes of Health, MIDAS = Migraine Disability Assessment, HIT = Headache Impact Test.

**Table 5:** Descriptive statistics across the harmonized health-related clinical variables in LIMBIC and TRACTS by TBI History (no TBI versus Mild TBI).

Characteristic	Harmony of scales	LIMBIC *N* = 2,868		TRACTS *N* = 905	
		mTBI *n* = 2,339*^1^*	No TBI *n* = 529*^1^*	mTBI *n* = 640*^1^*	No TBI *n* = 265*^1^*
**Health-related clinical**					
Sleep quality (PSQI)	●	10.7 (4.6); 11.0 (7.0,14.0)	8.8 (4.7); 8.5 (5.0,12.0)	11.2 (4.5); 12.0 (8.0,14.0)	9.4 (4.9); 9.0 (5.0,13.0)
Unknown		79	7	27	5
Body mass index (BMI)	◐ Derived from physical exam^a^ or self-reported height and weight^b^	30.4 (5.4); 29.7 (26.6,33.3)	29.7 (5.3); 29.2 (26.0,32.8)	29.4 (5.1); 28.7 (25.7,32.4)	29.3 (5.4); 28.8 (25.8,31.9)
Unknown		58	7	11	6
Blood pressure (Systolic)	●	128.3 (15.2); 127.0 (118.0,137.0)	127.7 (14.6); 127.0 (117.0,136.0)	121.1 (12.9); 121.0 (112.0,129.0)	119.7 (13.5); 120.0 (110.0,128.0)
Unknown		59	8	11	7
Blood pressure (Diastolic)	●	81.4 (10.2); 81.0 (75.0,88.0)	80.7 (9.4); 80.0 (74.0,87.0)	77.7 (9.8); 78.0 (71.0,84.0)	76.7 (10.2); 77.0 (70.0,83.0)
Unknown		59	8	11	7
Heart rate	●	67.6 (12.8); 66.0 (59.0,76.0)	66.3 (11.6); 65.0 (58.0,73.0)	70.4 (11.6); 68.5 (60.0,77.0)	68.6 (11.6); 68.0 (60.0,76.0)
Unknown		59	8	12	11
NSI 17: Fatigue, loss of energy	●				
None		380 (16.5%)	150 (28.5%)	134 (22.3%)	94 (36.6%)
Mild		707 (30.7%)	190 (36.1%)	143 (23.8%)	68 (26.5%)
Moderate		735 (31.9%)	121 (23.0%)	167 (27.8%)	45 (17.5%)
Severe		363 (15.7%)	50 (9.5%)	98 (16.3%)	32 (12.5%)
Very severe		121 (5.2%)	16 (3.0%)	59 (9.8%)	18 (7.0%)
Unknown		33	2	39	8

^1^Mean (SD); Median (Q1, Q3); n (%), ● = exact match, ◐ = adjustments needed to harmonize ^a^TRACTS; ^b^LIMBIC-CENC, PSQI = Pittsburgh Sleep Quality Index, NSI = Neurobehavioral Symptom Inventory.

*Social health.* Across measures of social support (DRRI) (LIMBIC: ω = 0.88; TRACTS: ω = 0.90) and life satisfaction (SWLS) (LIMBIC: ω = 0.90; TRACTS: ω = 0.93), reliability was acceptable and mean scores were similar across cohorts (Table 2). Regarding employment, full-time employment was the most common status, reported by 54.7% of LIMBIC and 49.7% of TRACTS and part-time employment was reported by 8.3% of LIMBIC and 12.8% of TRACTS.

*Sensory.* Dizziness impact (DHI-S) among mTBI participants was comparable in LIMBIC and TRACTS, with most participants reporting “little or no disability” (LIMBIC = 72.3%; TRACTS = 75.9%) and demonstrated good internal consistency (LIMBIC: ω = 0.84; TRACTS: ω = 0.90). Hearing-related handicap impact (HHIA-S) also showed high reliability (LIMBIC: ω = 0.90; TRACTS: ω = 0.93) and followed a similar pattern, with 54.7 and 31.2% of LIMBIC participants and 55.2 and 30.8% of TRACTS participants reporting “little or no disability” and “mild or moderate disability”, respectively. On the tinnitus impact measure, LIMBIC participants most frequently reported “little or no disability” (44.6%) and “moderate disability” (24.2%). Among TRACTS mTBI participants the most common responses were “little or no disability” (34.7%) and “mild disability” (37.8%). Across both cohorts, most participants with mTBI reported no symptoms on the individual items of the NSI (Table 3).

*Pain.* Pain intensity scores among mTBI participants diverged between cohorts (Table 4), with LIMBIC reporting a mean of 4.3 (SD = 2.4) and TRACTS a mean of 6.2 (SD = 2.4), which represents a clinically meaningful difference between the groups. On the Headache Disability Scale, a higher proportion of LIMBIC participants reported “severe disability” (37.2%) on the headache impact measure, whereas the most common response in TRACTS was “little or no disability” (48.9%).

*Health-related Clinical.* Sleep quality (PSQI) demonstrated acceptable reliability (LIMBIC: ω = 0.79; TRACTS: ω = 0.79) and average scores were similar in LIMBIC and TRACTS (Table 5). Physical health indicators revealed minor differences (which were not statistically tested). The average body mass index (BMI), systolic and diastolic blood pressure and heart rate were broadly comparable. Fatigue from the single item on the NSI is also presented in Table 5. Among those in LIMBIC with mTBI a slightly higher proportion endorsed mild and moderate fatigue (30.7, 31.9%) compared to 23.8% (mild) and 27.8% (moderate) in TRACTS.

### Principle component analysis results

**Figure 2:**
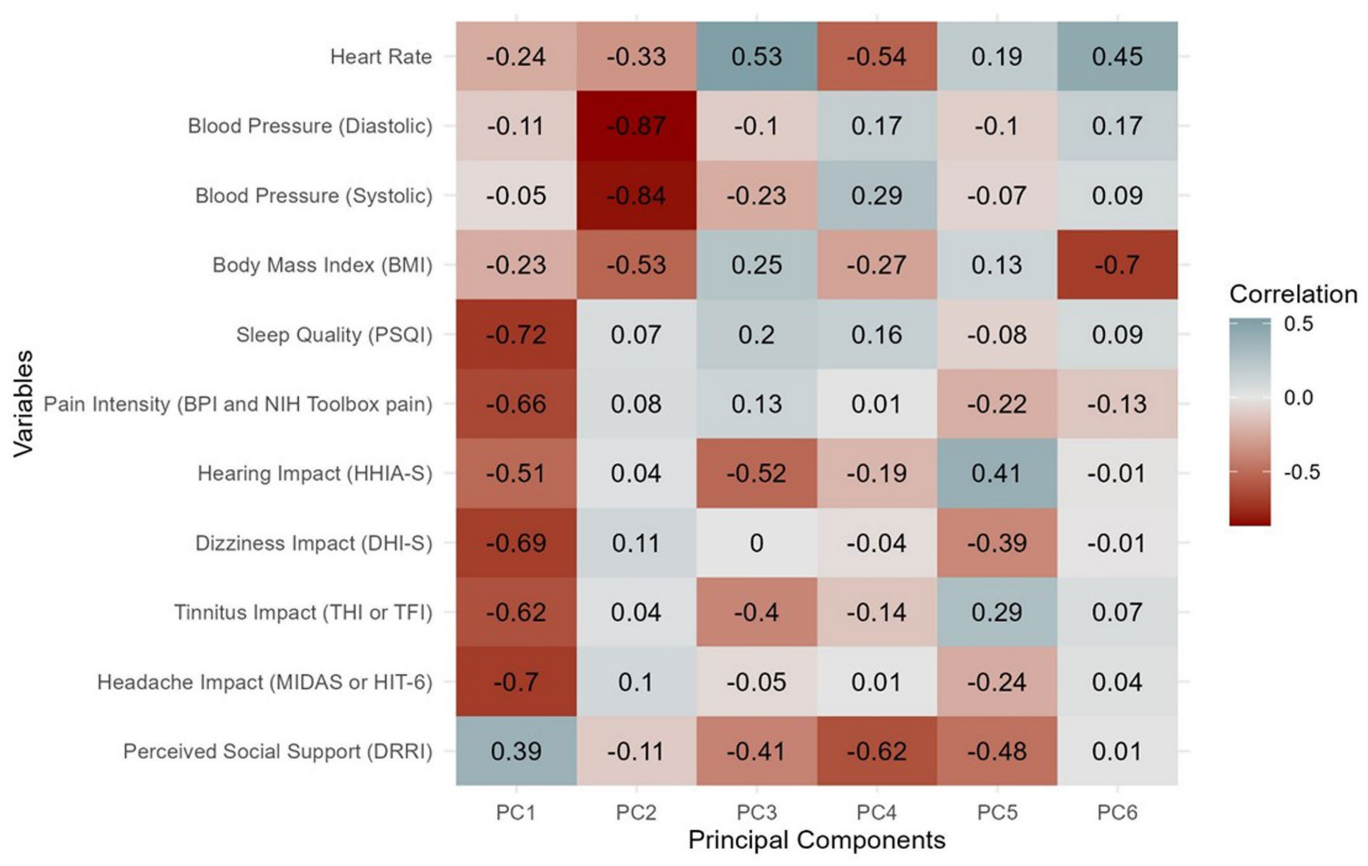
Correlations between variables and principal components. Heatmap of Pearson correlations between observed variables and principal components. Values represent the correlation between each variable’s scores and the component scores across participants. Correlation strength was interpreted as weak (|*r*| = 0.1–0.3), moderate (|*r*| = 0.4–0.6), and strong (|*r*| = 0.7–1.0). Positive and negative correlations are shown in blue and red, respectively. Color intensity reflects the magnitude of the correlation, with greater intensity indicating a stronger correlation. PC1 showed moderate-strong correlations with self-reported symptoms and weak-moderate correlation with perceived social support, while PC2 showed moderate-strong correlations with objective physiological measures.

**Figure 3:**
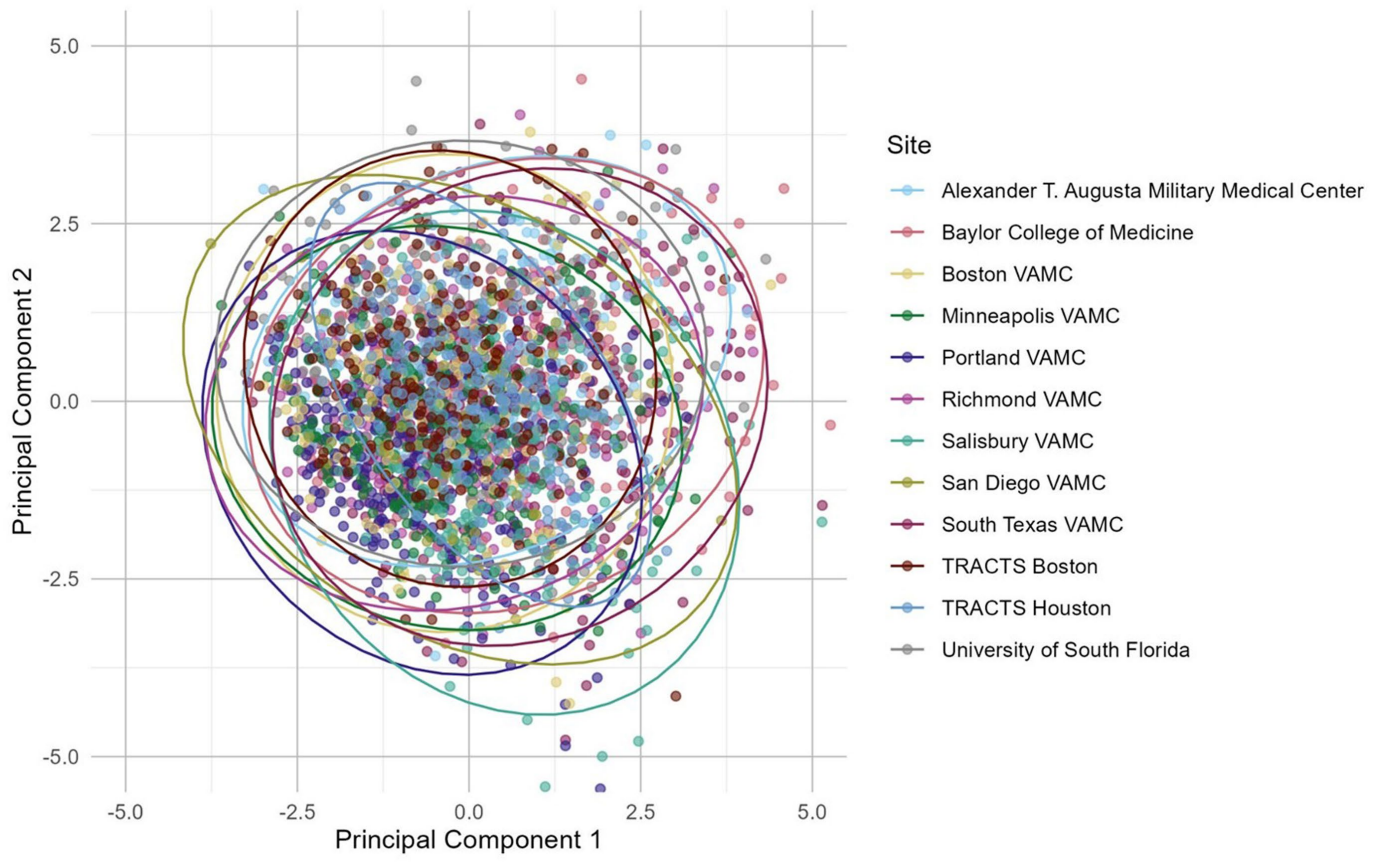
PCA score plot by site. Score plot of the first two principal components by site with each point representing a participant. Ellipses represent 95% confidence regions for each site. Overlapping points and ellipses across sites suggests similar multivariate distributions, indicating minimal site-level clustering.

The first six principal components explained 76% of the total variation in the data and were retained for exploratory analysis. The first principal component (PC1) showed strong negative correlations with PSQI global score, pain rating, HHIA-S total score, DHI-S total score, impact of Tinnitus, and impact of headaches and a weak to moderate positive correlation with DRRI social support. The second principal component (PC2) had a strong negative correlation with blood pressure and a moderate negative correlation with BMI (Figure 2). These correlations suggest PC1 and PC2 represent self-reported health outcomes and objective physiological measurements, respectively. Higher PC1 scores reflect lower symptom burden and greater perceived social support, and higher PC2 scores correspond to lower blood pressure and BMI. The score plot revealed overlapping data points and confidence ellipses across all sites, indicating minimal multivariate site-level differences. This overlap suggests that the harmonized data is suitable for pooled analysis (Figure 3).

## Discussion

LIMBIC and TRACTS datasets offer numerous variables to gain a more detailed understanding of longitudinal outcomes related to social and physical health, while considering key clinical, demographic, TBI injury, and other exposure-related characteristics. The goal of this proof-of-concept paper was to describe the methods used to harmonize two large, deeply characterized, prospective longitudinal cohort studies of U.S. veterans and SMs with mTBI and present an overview of the harmonized social and physical health-related variables. Overall, we were able to directly harmonize multiple self-report measures of social well-being and indirectly harmonize other functional and demographic variables by recoding, aligning categorical levels, and categorizing scales based on established cut scores. While this initial concept paper focused solely on harmonizing baseline data, the included principles can be employed to harmonize longitudinal data and examine associations between TBI subgroups, including those with multiple mTBIs, and outcomes.

While many of the health-related and demographic variables have been analyzed independently in the TRACTS and LIMBIC studies, our ability to successfully harmonize many of these variables has broader impacts on research methodologies and clinical implications. First, data linkage and harmonization allow for the use of advanced statistical methods requiring large and diverse data sources, such as machine learning (ML) and other artificial intelligence (AI) approaches (38–40). By establishing harmonization methods with these large, representative data sources of veteran and SM populations, we can build on those methods to link and validate other data sources, such as electronic health record data or other important data sources such as the Million Veterans Project (MVP) and the Individual Longitudinal Exposure Record (ILER) (41, 42). While ML and AI methods can enable exploratory and predictive modeling, preliminary modeling is more rapidly established and accurate when robust harmonization and data validation is the basis for unsupervised model building. Finally, these methods also serve as an important basis for precision medicine advances benefiting veterans and SMs, particularly those with combat exposure and mTBI history. By combining smaller subsamples across multiple large data sources, more complex analyses can examine outcomes relevant to subgroups and interactions that are otherwise too small to achieve adequate statistical power. For example, many findings from mTBI studies are unable to adequately differentiate whether and how results vary based on PTSD status or exposure to traumatic events, particularly due to the overlapping symptoms between mTBI history and PTSD. Larger datasets with deep characterization can open doors to these more nuanced traditional analyses (e.g., covariate adjustments or subgroup analyses) and provide a solid base on which ML/AI methods can be applied for advanced predictive analytics. Beyond these examples, our rich data sources also offer unique and detailed data on imaging, biomarker, and other key outcomes that will likely also drive precision medicine approaches in the future. Our TBD project team is also harmonizing, analyzing, and disseminating data for those measures in a similar manner as the present paper (18). Though these initial proof-of-concept harmonization datasets will allow for deeper examinations of functional and other clinical outcomes than has been possible in the past, the possibilities are exponentially greater when full harmonization of the data sources builds on these proof-of-concept methods, as is planned for the near future.

Data harmonization is not without challenges; there is often an information cost of harmonizing data without an exact match across studies (43). Our team sought to balance maximum information retention, with clinically meaningful harmonized data, resulting in a tailored, clean, dataset that is ready for traditional biostatistical analysis. In future research, we will also take a latent variable approach, which allows different measures of the same construct to be retained and processed through advanced analytics. Both approaches have relative strengths and weaknesses, but by openly describing data harmonization decisions we can support transparent and accurate use of data resources like TBD [(44); see Supplementary Table 1 for our Data Dictionary]. Thus, even these preliminary findings have value in establishing the evidence base for TBD and making way for more advanced analyses. Although the larger goal of the TBD program is to create a single, publicly available integrated dataset for deeper ML/AI analyses (40), even the more limited proof-of-concept data sources outlined in the present study can provide new clinical insights. For example, the present study included the first robust comparison of two independent and largescale data sources on social and physical health outcomes in veterans and SMs exposed to combat and mTBI. This harmonization process allowed us to validate the two studies against each other, finding robust evidence of similarities in demographics and health-related outcomes across studies and sites. In addition, exploratory PCA of the harmonized data set supported pooling of the data and revealed multivariate patterns consistent with established clinical relationships. For example, measures of symptom burden and social support loaded in opposite directions on the primary component, reflecting the expected inverse relationship between higher symptom burden and lower perceived social support. Where we did find differences (e.g., sensory symptoms), we can hypothesize why these differences exist across the studies and conduct exploratory analyses when data are available, or design follow-up data collection via subsequent research studies. Finally, this harmonized data set can provide an opportunity for identifying the clinically meaningful, minimum data required to inform evidence-based clinical care for veterans and SMs with and without history of TBI.

Several researchers have initiated work to allow for the analyses of the extensive TBI research data available publicly in the Federal Interagency Brain Injury Registry (FITBIR), yet these efforts are nascent. To optimally address the present knowledge gaps and harmonize largescale, multi-modal data from varied sources, well-planned and reproducible standardization, curation, and dissemination are necessary to enable clinically salient analyses. The lessons learned from this TBD proof of concept program *set the stage* for future researchers insights into the challenges and opportunities associated with such harmonization and help identify paths forward in these activities. The power of establishing large, well-harmonized datasets across parallel cohorts of research participants can be found in the increased generalizability of findings (i.e., enhanced external validity) and the ability to use advanced statistical approaches (i.e., multivariate modeling, subgroup analyses) that can increase the ability to detect clinical phenotypes to be applied in precision medicine approaches to TBI outcomes and care.

*Limitations, future directions, and clinical implications*: While the TBD program has demonstrated a successful proof of concept approach to the harmonization of these two large datasets, there are some limitations to the approach. Despite the overall similarities of the LIMBIC and TRACTS studies, variations in study eligibility and sample characteristics (e.g., recruitment methods, site locations, time post-injury, number of deployments, and survey administration skip logic) may explain the observed differences in some measures between studies. In this analysis, differences in TBI severity eligibility (mild only for LIMBIC; all severity categories for TRACTS) were mitigated by including only TRACTS participants with mTBI. Importantly, participants with no TBI history were included in both studies. Regarding recruitment methods, while TRACTS primarily recruits a community-based veteran sample with limited recruitment from VA clinics, LIMBIC recruited from the community and broader post-deployment population registered for care at each site. Future directions include both highlighting the data elements that were not harmonizable to help guide ongoing or new research activities in this area, and identifying the specific work required to streamline the harmonization process. These efforts will lead to enhanced data collection targeting the use of standardized questionnaires and common data elements and the secondary integration process to optimize efficiency and accuracy. Most of the harmonized measures used are readily accessible in clinical and research settings and could be utilized in a standard clinical assessment battery. This would allow for identification of care needs and/or characterizing phenotypes for personalized clinical care pathways along with tracking of improvement or decline over time.

## Data Availability

The datasets presented in this study can be found in online repositories. The names of the repository/repositories and accession number(s) can be found at: The Total Brain Diagnostics database is not currently publicly available; however, TRACTS and LIMBIC data are shared with the FITBIR database, publicly accessible at https://fitbir.nih.gov/content/submitted-data.
