# Supplementary figures and images for: Harmonization of social and physical health measures across prospective clinical studies of combat exposed service members and veterans: the total brain diagnostics program
Source: Front Neurol. 2026 Jul 8;17:1799509. doi: 10.3389/fneur.2026.1799509 (PMC13389360; doi:10.3389/fneur.2026.1799509)

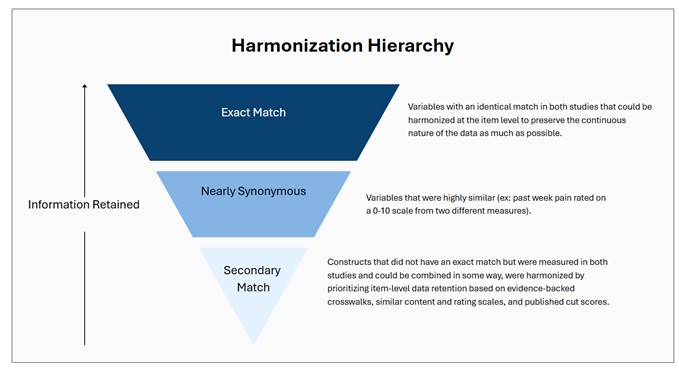

Supplement: Supplementary file 1 [file Image_1.jpg]
