# Supplementary material for: Harmonization of social and physical health measures across prospective clinical studies of combat exposed service members and veterans: the total brain diagnostics program
Source: Front Neurol. 2026 Jul 8;17:1799509. doi: 10.3389/fneur.2026.1799509 (PMC13389360; doi:10.3389/fneur.2026.1799509)
Supplement: Supplementary file 2 [file Table_1.docx]

**Supplemental Table 1. Data Dictionary**

| **Domain** | **Variable Name** | **Title** | **Definition** | **Variable Type** | **Permissible Values** | **Value Labels** | **Harmonization Type** | **TRACTS Variable Name** | **TRACTS Variable Harmonization** | **TRACTS Measure Type** | **LIMBIC Variable Name** | **LIMBIC Variable Harmonization** | **LIMBIC Measure Type** |
| --- | --- | --- | --- | --- | --- | --- | --- | --- | --- | --- | --- | --- | --- |
| DEMOG | age | Age | Age in years | Integer | 17 to 125 |  | Direct | BG_AGE |  | Self-report | DEMOGAGEYEARS |  | Self-report |
| DEMOG | race_white | Race: White | Participant Identified Race as White | Binary | 0;1 | No;Yes | Partial match | BG_RACE_White; ; BG_RACE_AmIndian; BG_RACE_Asian; BG_RACE_PacificIsland; BG_RACE_Other; BG_RACE_OtherSpec; BG_RACE_Unknown | Used TRACTS variable, no modifications | Self-report | DEMOGWHITE | Patient selected each race that applied. Assumed that missing values are "No". | Self-report |
| DEMOG | race_black | Race: Black or African American | Participant Identified Race as Black or African American | Binary | 0;1 | No;Yes | Partial match | BG_RACE_Black | Used TRACTS variable, no modifications | Self-report | DEMOGBLKAFAMER | Patient selected each race that applied. Assumed that missing values are "No". | Self-report |
| DEMOG | race_am_indian | Race: American Indian or Alaska Native | Participant Identified Race as American Indian or Alaska Native | Binary | 0;1 | No;Yes | Partial match | BG_RACE_AmIndian | Used TRACTS variable, no modifications | Self-report | DEMOGINDIANALASKA | Patient selected each race that applied. Assumed that missing values are "No". | Self-report |
| DEMOG | race_asian | Race: Asian | Participant Identified Race as Asian | Binary | 0;1 | No;Yes | Partial match | BG_RACE_Asian | Used TRACTS variable, no modifications | Self-report | DEMOGASIAN | Patient selected each race that applied. Assumed that missing values are "No". | Self-report |
| DEMOG | race_pacific_island | Race: Native Hawaiian or Other Pacific Islander | Participant Identified Race as Native Hawaiian or Other Pacific Islander | Binary | 0;1 | No;Yes | Partial match | BG_RACE_PacificIsland | Used TRACTS variable, no modifications | Self-report | DEMOGPACIFICISLNDR | Patient selected each race that applied. Assumed that missing values are "No". | Self-report |
| DEMOG | race_unknown | Race: Unknown | Participant Identified Race as Unknown | Binary | 0;1 | No;Yes | Partial match | BG_RACE_Unknown | Used TRACTS variable, no modifications | Self-report | DEMOGRACEOTHNODNTKNWREFUSED | Patient selected each race that applied. Assumed that missing values are "No". | Self-report |
| DEMOG | race_other | Race: Other | Participant Identified Race as Other | Binary | 0;1 | No;Yes | Partial match | BG_RACE_Other | Only available in TRACTS data | Self-report | DEMOGRACEOTHNODNTKNWREFUSED | Patient selected each race that applied. Assumed that missing values are "No". | Self-report |
| DEMOG | race_refused | Race: Refused to Answer | Participant Identified Race as Refused to Answer | Binary | 0;1 | No;Yes | Partial match | BG_RACE_White; BG_RACE_Black; BG_RACE_AmIndian; BG_RACE_Asian; BG_RACE_PacificIsland; BG_RACE_Other; BG_RACE_OtherSpec; BG_RACE_Unknown | Used TRACTS variable, no modifications | Self-report | DEMOGRACEOTHNODNTKNWREFUSED | Patient selected each race that applied. Assumed that missing values are "No". | Self-report |
| DEMOG | race_other_spec | Race: Other, Specified | Participant Specified Another Race | String |  |  | Single study | BG_RACE_Other_Spec | Only available in TRACTS data |  |  |  |  |
| DEMOG | ethnicity | Ethnicity | Participants's Ethnicity | Categorical | Hispanic or Latino Origin or Descent;Not of Hispanic or Latino Origin or Descent;Unknown;Refused |  | Partial match | BG_ETHN_Hispanic | Used TRACTS variable, no modifications | Self-report | DEMOGETHNICITY | Harmonized category values with TRACTS | Self-report |
| DEMOG | sex | Sex | Participant's sex | Categorical | Male;Female;Refused |  | Partial match | BG_GEND | Only contained values Man and Woman. Harmonized Man and Woman to Male and Female. | Self-report | GENDERTYP | Changed values from Man/Woman to Male/Female | Self-report |
| TBI_Subject | tbi_pt_yn | TBI Y/N | Indicator of whether the subject has had a concussion or TBI | Categorical | 0;1 | No;Yes | Partial match | LifetimeTBI | Used TRACTS variable, no modifications | Self-report | NumOfPOSITIVETBIs | Count >= 1 then 1 Else 0 | Self-report |
| FUNC | drri_social_support1 | DRRI Social Support Item 1 | The American people made me feel at home when I returned. | Integer | 1 to 5 | Strongly disagree;Somewhat disagree;Neither agree nor disagree;Somewhat agree;Strongly agree | Direct | DRRI_O1 - DRRI_O10 |  | Self-report | DRRI2OMADEMEFEELATHOME |  | Self-report |
| FUNC | drri_social_support2 | DRRI Social Support Item 2 | When I returned, people made me feel proud to have served my country in the Armed Forces. | Integer | 1 to 5 | Strongly disagree;Somewhat disagree;Neither agree nor disagree;Somewhat agree;Strongly agree | Direct | DRRI_O1 - DRRI_O10 |  | Self-report | DRRI2OMADEMEFEELPROUD |  | Self-report |
| FUNC | drri_social_support3 | DRRI Social Support Item 3 | My family members/friends made me feel better when I am down. | Integer | 1 to 5 | Strongly disagree;Somewhat disagree;Neither agree nor disagree;Somewhat agree;Strongly agree | Direct | DRRI_O1 - DRRI_O10 |  | Self-report | DRRI2OMADEMEFEELBETTER |  | Self-report |
| FUNC | drri_social_support4 | DRRI Social Support Item 4 | I can go to family members/friends when I need good advice. | Integer | 1 to 5 | Strongly disagree;Somewhat disagree;Neither agree nor disagree;Somewhat agree;Strongly agree | Direct | DRRI_O1 - DRRI_O10 |  | Self-report | DRRI2OCANGOTOFAMILYFRIENDS |  | Self-report |
| FUNC | drri_social_support5 | DRRI Social Support Item 5 | My family/friends understand what I have been through in the Armed Forces. | Integer | 1 to 5 | Strongly disagree;Somewhat disagree;Neither agree nor disagree;Somewhat agree;Strongly agree | Direct | DRRI_O1 - DRRI_O10 |  | Self-report | DRRI2OFAMILYFRIENDSUNDERSTAND |  | Self-report |
| FUNC | drri_social_support6 | DRRI Social Support Item 6 | There are family/friends with whom I can talk about my deployment experiences. | Integer | 1 to 5 | Strongly disagree;Somewhat disagree;Neither agree nor disagree;Somewhat agree;Strongly agree | Direct | DRRI_O1 - DRRI_O10 |  | Self-report | DRRI2OFAMILYFRIENDSTOSHARE |  | Self-report |
| FUNC | drri_social_support7 | DRRI Social Support Item 7 | My family members/friends would lend me money if I needed it. | Integer | 1 to 5 | Strongly disagree;Somewhat disagree;Neither agree nor disagree;Somewhat agree;Strongly agree | Direct | DRRI_O1 - DRRI_O10 |  | Self-report | DRRI2OFAMILYFRIENDSTOLEND |  | Self-report |
| FUNC | drri_social_support8 | DRRI Social Support Item 8 | My family members/friends would help me move if I needed help. | Integer | 1 to 5 | Strongly disagree;Somewhat disagree;Neither agree nor disagree;Somewhat agree;Strongly agree | Direct | DRRI_O1 - DRRI_O10 |  | Self-report | DRRI2OFAMILYFRIENDSTOMOVE |  | Self-report |
| FUNC | drri_social_support9 | DRRI Social Support Item 9 | If I were unable to attend to daily chores, there is someone to help me. | Integer | 1 to 5 | Strongly disagree;Somewhat disagree;Neither agree nor disagree;Somewhat agree;Strongly agree | Direct | DRRI_O1 - DRRI_O10 |  | Self-report | DRRI2OFAMILYTOHELPDAILYCHORES |  | Self-report |
| FUNC | drri_social_support10 | DRRI Social Support Item 10 | When I am ill, family members/friends will help out until I am well. | Integer | 1 to 5 | Strongly disagree;Somewhat disagree;Neither agree nor disagree;Somewhat agree;Strongly agree | Direct | DRRI_O1 - DRRI_O10 |  | Self-report | DRRI2OFAMILYFRIENDSTOGETWELL |  | Self-report |
| FUNC | drri_social_support_tot | DRRI Social Support Total Score | Social Support Total Score | Integer | 10 to 50 |  | Direct | DRRI_O1 - DRRI_O10 |  | Self-report | DRRI2_SOCIAL_TOT |  | Self-report |
| FUNC | employment | Employment status | Whether the participant is employed (full or part time) or unemployed | Categorical | Not Working;Part-time;Full-time |  | Partial match | BG_WORK | BG_WORK: current working status, part or full time | Self-report | TBIMSEDCOMPEMPLOYED; TBIMSEDPAIDHOURSPERWK | If endorsed TBIMSEDCOMPEMPLOYED = No then Not working. Else if TBIMSEDPAIDHOURSPERWK >= 35 then Full-time. Else Part-time. | Self-report |
| FUNC | swls1 | Satisfaction with Life Scale Item 1 | In most ways my life is close to my ideal. | Integer | 1 to 7 | Strongly disagree;Disagree;Slightly disagree;Neither agree nor disagree;Slightly agree;Agree;Strongly agree | Direct | SLS_LifeIdeal |  | Self-report | SWLSLIFCLOSIDLSCORE |  | Self-report |
| FUNC | swls2 | Satisfaction with Life Scale Item 2 | The conditions of my life are excellent. | Integer | 1 to 7 | Strongly disagree;Disagree;Slightly disagree;Neither agree nor disagree;Slightly agree;Agree;Strongly agree | Direct | SLS_LifeCondExcellent |  | Self-report | SWLSLIFCONDEXCLLNCSCORE |  | Self-report |
| FUNC | swls3 | Satisfaction with Life Scale Item 3 | I am satisfied with life. | Integer | 1 to 7 | Strongly disagree;Disagree;Slightly disagree;Neither agree nor disagree;Slightly agree;Agree;Strongly agree | Direct | SLS_LifeSatisfied |  | Self-report | SWLSLIFSATFCTNSCORE |  | Self-report |
| FUNC | swls4 | Satisfaction with Life Scale Item 4 | So far I have gotten the important things I want in life. | Integer | 1 to 7 | Strongly disagree;Disagree;Slightly disagree;Neither agree nor disagree;Slightly agree;Agree;Strongly agree | Direct | SLS_LifeAchieve |  | Self-report | SWLSLIFACHVMNTSCORE |  | Self-report |
| FUNC | swls5 | Satisfaction with Life Scale Item 5 | If I could live my life over, I would change almost nothing. | Integer | 1 to 7 | Strongly disagree;Disagree;Slightly disagree;Neither agree nor disagree;Slightly agree;Agree;Strongly agree | Direct | SLS_NotChangeLife |  | Self-report | SWLSLIFCHNGSCORE |  | Self-report |
| FUNC | swls_tot | Satisfaction with Life Scale Total Score | Satisfaction with Life Scale Total Score | Integer | 0 to 3 |  | Direct | SLS_TOT |  | Self-report | SWLS_TOT |  | Self-report |
| FUNC | psqi_qlty | Pittsburgh Sleep Quality Index | Subjective Sleep Quality | Integer | 0 to 3 |  | Direct | PSQI_QLTY |  | Self-report | PSQI_SLPQUAL |  | Self-report |
| FUNC | psqi_laten | Pittsburgh Sleep Quality Index | Sleep Latency | Integer | 0 to 3 |  | Direct | PSQI_LAT |  | Self-report | PSQI_LATEN |  | Self-report |
| FUNC | psqi_dur | Pittsburgh Sleep Quality Index | Sleep Duration | Integer | 0 to 3 |  | Direct | PSQI_DUR |  | Self-report | PSQI_DURAT |  | Self-report |
| FUNC | psqi_hse | Pittsburgh Sleep Quality Index | Habitual Sleep Efficiency | Integer | 0 to 3 |  | Direct | PSQI_EFF |  | Self-report | PSQI_HSE |  | Self-report |
| FUNC | psqi_distb | Pittsburgh Sleep Quality Index | Sleep Disturbances | Integer | 0 to 3 |  | Direct | PSQI_DIST |  | Self-report | PSQI_DISTB |  | Self-report |
| FUNC | psqi_meds | Pittsburgh Sleep Quality Index | Frequency of Sleep Medication Use | Integer | 0 to 3 |  | Direct | PSQI_MEDS |  | Self-report | PSQI_MEDS |  | Self-report |
| FUNC | psqi_daydys | Pittsburgh Sleep Quality Index | Daytime Dysfunction | Integer | 0 to 3 |  | Direct | PSQI_DAYDYS |  | Self-report | PSQI_DAYDYS |  | Self-report |
| FUNC | psqi_global | Pittsburgh Sleep Quality Index | Global Score | Integer | 0 to 21 |  | Direct | PSQI_GLOBAL |  | Self-report | PSQI_TOT |  | Self-report |
| FUNC | pain_rating | Past week pain 0-10 |  | Integer | 0 to 10 |  | Partial match | BPI_ | 0-10 worst pain in the past week | Self-report | PAININTENSITY | 0-10 likert rating (last 7 days) | Self-report |
| FUNC | headache_yn | Does the participant have headaches? Y/N |  | Binary | 0;1 | No;Yes | Partial match | MIDAS_HEADACHE_YN | If participant answered yes to screening question, then coded as 1. If participant answered no to screening question, then coded as 0. If participant did not responded to screening question, then the value is NA. | Self-report | HIT6 | Little or No Impact: 49 or less Some Impact: 50-55 Substantial Impact : 56-59 Severe Impact: 60-78 | Self-report |
| FUNC | headache_disability | Impact of headache on functioning |  | Categorical | Not Administered; Little or No Disability; Mild Disability; Moderate Disability; Severe Disability |  | Partial match | MIDAS_TOT | Little or No Disability: 0-5 Mild Disability: 6-10 Moderate Disability: 11-20 Severe Disability: 21+ Not administered: headache_yn = 0 | Self-report | HIT6 | Not Administered: headache_yn = 0 Little or No Disability: 49 or less Mild Disability: 50-55 Moderate Disability: 56-59 Severe Disability: 60-78 | Self-report |
| FUNC | tinnitus_yn | Does the participant have tinnitus? Y/N |  | Binary | 0;1 | No;Yes | Direct | THI_Screen_1; THI_Screen_2; THI_Screen_3 | If participant answered yes to any of the three screening questions, then coded as 1. If participant answered no to all of the screening questions, then coded as 0. If participant did not responded to screening questions, then the value is NA. | Self-report | TFIEXPERIENCE | Experience tinnitus in the past week? | Self-report |
| FUNC | tinnitus_disability | Impact of tinnitus on functioning |  | Categorical | Not Administered;Little or No Disability; Mild Disability; Moderate Disability; Severe Disability;Extremely Severe Disability |  | Partial match | THI_TOT | 0-16: Slight or no handicap (Grade 1) 18-36: Mild handicap (Grade 2) 38-56: Moderate handicap (Grade 3) 58-76: Severe handicap (Grade 4) 78-100: Extremely severe handicap (Grade 5) Not administered: tinnitus_yn = 0 | Self-report | TFI_SCORE | Not Administered: tinnitus_yn = 0 Little or No Disability: 0-17 Mild Disability: 18-31 Moderate Disability: 32-53 Severe Disability: 54-72 Extremely Severe Disability: 73-100 | Self-report |
| FUNC | dizziness_yn | Does the participant have dizziness? Y/N | Lately have you been having a problem with dizziness or unsteadiness? | Binary | 0;1 | No;Yes | Single Study | THI_TOT | If THI_TOT was equal to zero or greater, coded as 1. If THI_TOT was missing, then coded as 0. | Self-report | DHISDIZZYUNSTEADY |  | Self-report |
| FUNC | dizziness_disability | DHI-S |  | Categorical | Little or No Disability; Mild Disability; Moderate Disability; Severe Disability;Extremely Severe Disability |  | Partial match | DHI_Depressed; DHI_Sidewalk; DHI_Concentrate; DHI_DarkHouse; DHI_BendOver; DHI_RestrictTravel; DHI_InterfereResp; DHI_AfraidLeaving; DHI_Embarassed; DHI_SocialActivities | Sum of DHI variables (DHI_Depressed - DHI_SocialActivities) and categorized as: Little or No Disability: 0-10 or hearing_problem_yn=0 Mild Disability: 11-20 Moderate Disability: 21-30 Severe Disability: 31-40 | Self-report | DHIS_TOT | Little or No Disability: 0-10 or hearing_problem_yn=0 Mild Disability: 11-20 Moderate Disability: 21-30 Severe Disability: 31-40 | Self-report |
| FUNC | dhis_depressed | DHI-S | Because of your problem, do you feel depressed? | Categorical | 0;2;4 | No;Sometimes;Yes | Direct | DHI_Depressed |  | Self-report | DHISDEPRESSED |  | Self-report |
| FUNC | dhis_sidewalk | DHI-S | Does walking down a sidewalk increase your problem? | Categorical | 0;2;4 | No;Sometimes;Yes | Direct | DHI_Sidewalk |  | Self-report | DHISSIDEWALKINCREASEPROB |  | Self-report |
| FUNC | dhis_concentrate | DHI-S | Because of your problem, is it difficult to concentrate? | Categorical | 0;2;4 | No;Sometimes;Yes | Direct | DHI_Concentrate |  | Self-report | DHISDIFFICULTCONCENTRATE |  | Self-report |
| FUNC | dhis_darkhouse | DHI-S | Because of your problem, is it difficult for you to walk around your house in the dark? | Categorical | 0;2;4 | No;Sometimes;Yes | Direct | DHI_DarkHouse |  | Self-report | DHISWALKDARK |  | Self-report |
| FUNC | dhis_bendover | DHI-S | Does bending over increase your problem? | Categorical | 0;2;4 | No;Sometimes;Yes | Direct | DHI_BendOver |  | Self-report | DHISBENDINCREASEPROB |  | Self-report |
| FUNC | dhis_restricttravel | DHI-S | Because of your problem, do you restrict your travel for business or recreation? | Categorical | 0;2;4 | No;Sometimes;Yes | Direct | DHI_RestrictTravel |  | Self-report | DHISRESTRICTTRAVEL |  | Self-report |
| FUNC | dhis_interfereresp | DHI-S | Does your problem interfere with your job or household responsibilities? | Categorical | 0;2;4 | No;Sometimes;Yes | Direct | DHI_InterfereResp |  | Self-report | DHISINTERFEREJOBHOUSE |  | Self-report |
| FUNC | dhis_afraidleaving | DHI-S | Because of your problem, are you afraid to leave your home with-out having someone with you? | Categorical | 0;2;4 | No;Sometimes;Yes | Direct | DHI_AfraidLeaving |  | Self-report | DHISAFRAIDTOLEAVEHOME |  | Self-report |
| FUNC | dhis_embarrassed | DHI-S | Because of your problem, have you ever been embarrassed in front of others? | Categorical | 0;2;4 | No;Sometimes;Yes | Direct | DHI_Embarrassed |  | Self-report | DHISEMBARRASSED |  | Self-report |
| FUNC | dhis_socialactivities | DHI-S | Does your problem significantly restrict your participation in socialactivities such as going out to dinner, going to movies, dancing orto parties? | Categorical | 0;2;4 | No;Sometimes;Yes | Direct | DHI_SocialActivities |  | Self-report | DHISRESTRICTSOCIALACTIVITY |  | Self-report |
| FUNC | dhis_tot | DHI-S | Total of 10 DHIS Items | Integer | 0 to 40 |  | Partial match | DHI_Depressed; DHI_Sidewalk; DHI_Concentrate; DHI_DarkHouse; DHI_BendOver; DHI_RestrictTravel; DHI_InterfereResp; DHI_AfraidLeaving; DHI_Embarassed; DHI_SocialActivities | Sum of DHI variables (DHI_Depressed - DHI_SocialActivities) |  | DHIS_TOT |  | Self-report |
| FUNC | hearing_problem_yn | HHIA-S lead in question | Have you experienced problems with hearing lately? If "no" HHIAS not administered | Binary | 0;1 | No;Yes | Single Study |  |  | Self-report | HHIASPROBLEMWITHHEARING |  | Self-report |
| FUNC | hearing_disability | HHIA-S |  | Categorical | Little or No Disability; Mild to Moderate Disability; Severe Disability |  | Partial match | HHIA_Embarrassed; HHIA_FrustratedFamily; HHIA_UnderstandWork; HHIA_FeelHandicapped; HHIA_VisitFriends; HHIA_Movies; HHIA_ArgueFamily; HHIA_RadioTV; HHIA_Socialize; HHIA_Restaurant | Sum of HHIA variables (HHIA_Embarrassed - HHIA_Restaurant) and categorized as: Little or No Disability: 0-8 or hearing_problem_yn=0 Mild to Moderate Disability: 9-25 Severe Disability: 26-40 | Self-report | HHIAS_TOT | Little or No Disability: 0-8 or hearing_problem_yn=0 Mild to Moderate Disability: 9-25 Severe Disability: 26-40 | Self-report |
| FUNC | hhias_embarrassed | HHIA-S | Does a hearing problem cause you to feel embarrassed when meeting new people? | Categorical | 0;2;4 | No;Sometimes;Yes | Direct | HHIA_Embarrassed |  | Self-report | HHIASEMBARASSEDNEWPEOPLE |  | Self-report |
| FUNC | hhias_frustrated_family | HHIA-S | Does a hearing problem cause you to feel frustrated when talking to members of your family? | Categorical | 0;2;4 | No;Sometimes;Yes | Direct | HHIA_FrustratedFamily |  | Self-report | HHIASFEELFRUSTRATED |  | Self-report |
| FUNC | hhias_understand_work | HHIA-S | Does a hearing problem cause you difficulty hearing/understanding co--worker, clients or customers? | Categorical | 0;2;4 | No;Sometimes;Yes | Direct | HHIA_UnderstandWork |  | Self-report | HHIASDIFFICULTYUNDERSTANDING |  | Self-report |
| FUNC | hhias_feel_handicapped | HHIA-S | Do you feel handicapped by a hearing problem? | Categorical | 0;2;4 | No;Sometimes;Yes | Direct | HHIA_FeelHandicapped |  | Self-report | HHIASFEELHANDICAPPED |  | Self-report |
| FUNC | hhias_visit_friends | HHIA-S | Does a hearing problem cause you difficulty when visiting friends, relatives or neighbors? | Categorical | 0;2;4 | No;Sometimes;Yes | Direct | HHIA_VisitFriends |  | Self-report | HHIASDIFFICULTYVISITING |  | Self-report |
| FUNC | hhias_movies | HHIA-S | Does a hearing problem cause you difficulty in the movies or theater? | Categorical | 0;2;4 | No;Sometimes;Yes | Direct | HHIA_Movies |  | Self-report | HHIASDIFFICULTYINMOVIES |  | Self-report |
| FUNC | hhias_argue_family | HHIA-S | Does a hearing problem cause you to have arguments with family members? | Categorical | 0;2;4 | No;Sometimes;Yes | Direct | HHIA_ArgueFamily |  | Self-report | HHIASARGUMENTSFAMILY |  | Self-report |
| FUNC | hhias_radio_tv | HHIA-S | Does a hearing problem cause you difficulty when listening to TV or radio? | Categorical | 0;2;4 | No;Sometimes;Yes | Direct | HHIA_RadioTV |  | Self-report | HHIASDIFFICULTYLISTENINGTV |  | Self-report |
| FUNC | hhias_socialize | HHIA-S | Do you feel that any difficulty with your hearing limits or hampers your personal or social life? | Categorical | 0;2;4 | No;Sometimes;Yes | Direct | HHIA_Socialize |  | Self-report | HHIASHAMPERPERSONALLIFE |  | Self-report |
| FUNC | hhias_restaurant | HHIA-S | Does a hearing problem cause you difficulty when in a restaurant with relatives or friends? | Categorical | 0;2;4 | No;Sometimes;Yes | Direct | HHIA_Restaurant |  | Self-report | HHIASDIFFICULTYRESTAURANT |  | Self-report |
| FUNC | hhias_tot | HHIA-S | HHIA-S Total Score | Integer | 0 to 40 |  | Partial match | HHIA_Embarrassed; HHIA_FrustratedFamily; HHIA_UnderstandWork; HHIA_FeelHandicapped; HHIA_VisitFriends; HHIA_Movies; HHIA_ArgueFamily; HHIA_RadioTV; HHIA_Socialize; HHIA_Restaurant | Sum of HHIA variables (HHIA_Embarrassed - HHIA_Restaurant) | Self-report | HHIAS_TOT |  | Self-report |
| FUNC | nsi1 | Self-reported neurocognitive symptoms | NSI 1: Feeling Dizzy | Integer | 0 to 4 | None;Mild;Moderate;Severe;Very Severe | Direct | NSI_1 |  | Self-report | NSIDIZSCORE | Data collection form has mbass in NSI | Self-report |
| FUNC | nsi2 | Self-reported neurocognitive symptoms | NSI 2: Loss of balance | Integer | 0 to 4 | None;Mild;Moderate;Severe;Very Severe | Direct | NSI_2 |  | Self-report | NSIBALNCIMPRMNTSCORE | Data collection form has mbass in NSI | Self-report |
| FUNC | nsi3 | Self-reported neurocognitive symptoms | NSI 3: Poor coordination, clumsy | Integer | 0 to 4 | None;Mild;Moderate;Severe;Very Severe | Direct | NSI_3 |  | Self-report | NSICOORDNTNIMPRMNTSCORE | Data collection form has mbass in NSI | Self-report |
| FUNC | nsi5 | Self-reported neurocognitive symptoms | NSI 5: Nausea/vomiting | Integer | 0 to 4 | None;Mild;Moderate;Severe;Very Severe | Direct | NSI_5 |  | Self-report | NSINAUSSCORE | Data collection form has mbass in NSI | Self-report |
| FUNC | nsi6 | Self-reported neurocognitive symptoms | NSI 6: Vision problems, blurring, trouble seeing | Integer | 0 to 4 | None;Mild;Moderate;Severe;Very Severe | Direct | NSI_6 |  | Self-report | NSIVISNIMPRMNTSCORE | Data collection form has mbass in NSI | Self-report |
| FUNC | nsi7 | Self-reported neurocognitive symptoms | NSI 7: Sensitivity to light | Integer | 0 to 4 | None;Mild;Moderate;Severe;Very Severe | Direct | NSI_7 |  | Self-report | NSISENSTVLGTSCORE | Data collection form has mbass in NSI | Self-report |
| FUNC | nsi9 | Self-reported neurocognitive symptoms | NSI 9: Sensitivity to noise | Integer | 0 to 4 | None;Mild;Moderate;Severe;Very Severe | Direct | NSI_9 |  | Self-report | NSISENSTVNOISSCORE | Data collection form has mbass in NSI | Self-report |
| FUNC | nsi10 | Self-reported neurocognitive symptoms | NSI 10: Numbness or tingling on parts of my body | Integer | 0 to 4 | None;Mild;Moderate;Severe;Very Severe | Direct | NSI_10 |  | Self-report | NSINUMBNSSIMPRMNTSCORE | Data collection form has mbass in NSI | Self-report |
| FUNC | nsi11 | Self-reported neurocognitive symptoms | NSI 11: Change in taste and/or smell | Integer | 0 to 4 | None;Mild;Moderate;Severe;Very Severe | Direct | NSI_11 |  | Self-report | NSITASTIMPRMNTSCORE | Data collection form has mbass in NSI | Self-report |
| FUNC | nsi12 | Self-reported neurocognitive symptoms | NSI 12: Loss of appetite or increase appetite | Integer | 0 to 4 | None;Mild;Moderate;Severe;Very Severe | Direct | NSI_12 |  | Self-report | NSIAPPTITIMPRMNTSCORE | Data collection form has mbass in NSI | Self-report |
| FUNC | nsi17 | Self-reported neurocognitive symptoms | NSI 17: Fatigue, loss of energy, getting tired easily, drowsiness | Integer | 0 to 4 | None;Mild;Moderate;Severe;Very Severe | Direct | NSI_17 |  | Self-report | NSIFATIGIMPRMNTSCORE | Data collection form has mbass in NSI | Self-report |
| FUNC | bmi | Body Mass Index |  | Decimal | 5 to 220 |  | Direct | MED_BMI |  | Objective | STOPBBANG_BMI |  | Objective |
| FUNC | bp_sys | Blood Pressure Systolic | Systolic Blood Pressure (Average standing and seated) | Integer | 20 to 300 |  | Direct | AVE_MED_BPSYS_ST; AVE_MED_BPDIA_ST | Systolic and diastolic Seated | Objective | BLDPRESSRSYSTMEASR | Systolic and diastolic Seated | Objective |
| FUNC | bp_dia | Blood Pressure Diastolic | Diastolic Blood Pressure (Average standing and seated) | Integer | 20 to 200 |  | Direct | AVE_MED_BPSYS_ST; AVE_MED_BPDIA_ST | Systolic and diastolic Seated | Objective | BLDPRESSRDIASTLMEASR | Systolic and diastolic Seated | Objective |
| FUNC | hr | Heart Rate | Seated Pulse | Integer | 25 to 220 |  | Direct | MED_PULSE_ST | standing | Objective | HEARTRATE |  | Objective |
